# Hospital Prevalence of Colorectal Cancer among Colonoscopy Recipients Attending a Tertiary Hospital in Oman: A Cross-Sectional Study

**DOI:** 10.1155/2020/5863126

**Published:** 2020-04-14

**Authors:** Alanoud F. Alsumait, Yahya M. Al-Farsi, Mostafa I. Waly, Issa S. Al-Qarshoobi, Samir Al-Adawi, Nawaf H. Albali, Mansour S. Al-Moundhri

**Affiliations:** ^1^Department of Family Medicine & Public Health, College of Medicine & Health Sciences, Sultan Qaboos University Muscat, Oman. P.O. Box 35, P.C. 123 Al-Khoudh, Muscat, Oman; ^2^Department of Food Sciences & Nutrition, College of Agriculture & Marine Sciences, Sultan Qaboos University, Muscat, Oman. P.O. Box 35, P.C. 123 Al-Khoudh, Oman; ^3^Department of Internal Medicine, College of Medicine & Health Sciences, Sultan Qaboos University Muscat, Oman. P.O. Box 35, P.C. 123 Al-Khoudh, Muscat, Oman; ^4^Department of Behavioral Medicine, College of Medicine & Health Sciences, Sultan Qaboos University, Muscat, Oman. P.O. Box 35, P.C. 123 Al-Khoudh, Oman; ^5^Field Epidemiology Training Program, Deputyship of Public Health, Ministry of Health Saudi Arabia, Riyadh, Saudi Arabia

## Abstract

**Purpose:**

Evidence from industrialized/developed countries showed that colorectal cancer (CRC) incidence rates have significantly dropped due to the widespread use of colonoscopy. In Arab countries, however, the CRC had been reported to have increased. Despite the concerted effort in the primary prevention and widespread use of colonoscopy, to our knowledge, there have been no reports of the prevalence rate of CRC among colonoscopy recipients from Oman. This study aims to explore the CRC prevalence estimates over selected sociodemographic characteristics among colonoscopy-recipients at a tertiary hospital in Oman over five years of follow-up. The regional variations in Oman were also examined in this study.

**Methods:**

This hospital-based cross-sectional study reviewed reports of colonoscopies performed over 5-years of retrospective follow-up at a tertiary hospital in Oman. CRC prevalence estimates were calculated over age, gender, governorate, and time of follow-up.

**Results:**

A total of 442 CRC cases were enumerated among 3701 colonoscopies, with an overall CRC prevalence estimate of 11.9 per 100 colonoscopies (95% CI: 10.9, 13.0). Gender-specific CRC prevalence was higher among males compared with females (13.3 vs. 10.5). Age-specific CRC prevalence increased with advancing age, from 2.8 among those less than 40 years of age to 26.5 among aged 70 years or more. Regional CRC prevalence was highest among residents in Batinah Governorate. Over the 5-years of follow-up, there was a slow rise in CRC prevalence with an annual increment of 0.59%.

**Conclusion:**

The study provides supportive evidence for a steady increase in CRC prevalence over age categories and years of follow-up and depicted the variations of gender-specific CRC prevalence estimates over increasing age categories. The study calls for timely formulation and adoption of national CRC screening programs centered on the colonoscopy use as primary prevention and maximizing its utilization and efficiency.

## 1. Introduction

Colorectal cancer (CRC) is the third most common cancer worldwide after lung and breast cancers with two-thirds of all colorectal cancers occurring in the more developed regions of the world [1]. It is predicted that about 2.4 million cases will be diagnosed annually worldwide by 2035 [2].

Colorectal cancer incidence is lower in developed compared with developing countries [3]. In 2014, the American Cancer Society released data showing colon cancer incidence rates to have dropped by 30% among people 50 years and older in the U.S [4]. This trend in the USA has been attributed to the widespread use of colonoscopy.

The situation is different in some of the emerging countries including those in the Arab world. Although the data reported for colorectal cancer are much lower than that of developed countries, the incidence of colorectal cancer in Arab countries has been on the rise in the past two decades [5–8]. This increase was attributed mainly to changes in lifestyle and dietary habits [9, 10].

In the Sultanate of Oman, a Middle Eastern Arab country with a population of 4.8 million, it has been shown for the year 2015 that the overall standardized global cancer incidence rate was 103.8 cases per 100,000 population, which was higher than what has been reported from Saudi Arabia and the UAE. CRC constituted about 11.2% of the total global cancer incidence. It was reported to be the second-most common cancer (10.95%) among males and the third-most among females (7.50%). According to the Oman National Cancer Registry 2015, age-standardized incidence rates (ASR) stood at 12 and 9.1 per 100,000 cases among men and women, respectively [11]. The majority of them were affected by adenocarcinoma, constituting about 80% of the total cases. As per the national registry, the CRC in Oman comprised of 9.0% of all-cause mortality in adult Omani males and 8.3% in females.

Colonoscopy is strongly recommended as a screening and early diagnostic tool, which dramatically benefits a reduction in CRC incidence and death rates in developed countries [12–14].

Colonoscopy is the most common diagnostic test in symptomatic individuals despite that computed tomography colonography provides a similarly sensitive, less invasive alternative to colonoscopy in patients presenting with symptoms suggestive of CRC [15]. Hence, given that colonoscopy permits removal/biopsy of the lesion, colonoscopy remains the gold standard for investigation of symptoms suggestive of CRC [16].

There is an indication that existing screening and early detection services for CRC are considered rudimentary in many Arab countries including Oman. Consequently, the presence of CRC cases is only detected in the late stages or at advance irreversible pathology [17].

To lay the groundwork for evidence-based policies for prevention, this study endeavored to explore the CRC prevalence estimates among colonoscopy-recipients at a tertiary hospital in Oman over five years of follow-up. The interrelated objectives of this study are as follows: (1) to describe the sociodemographic and clinical characteristics of CRC cases among colonoscopy recipients and (2) to provide CRC prevalence estimates concerning sociodemographic categories such as age, gender, governorate, and time of follow-up. To our knowledge, this is the first study to address the CRC prevalence among colonoscopy-recipients in the Arab region.

## 2. Methods

For the study, we conducted a cross-sectional study in a form of a retrospective review of all reports of colonoscopies conducted during the study period at Sultan Qaboos University Hospital (SQUH): a 570-bed, tertiary care teaching hospital in Muscat, Oman. Oman is divided into eleven governorates (muhafazah) and for brevity, and the governorates were lumped into 7 (Muscat, Batinah, Dakhliya, Sharqiya, Dhofar, Dhahira, and Buraimi) as these governorates are the most populated. Another reason for this was the fact that being a tertiary care hospital, SQUH also received a majority of its referrals from regional secondary and primary centers located in the aforementioned governorates. The study has been conducted for a period between January 2014 and December 2018.

### 2.1. Participants

Allowing an error rate of 2.5%, a level of significance (type 1 error) of 5% and 95% confidence interval and with a priori estimate of 10% prevalence of CRC among colonoscopy recipients as primary prevention [18], the least odds ratio of 2.5 was employed for this study. The Open Epi was used to calculate the sample size. The calculation indicated that a sample size of 380 was required to achieve a power of 80% in this study.

The study included participant colonoscopies performed at the Day Care Unit on adults aged 18 years and above of similar ethnicity, culture, and quality of care. The colonoscopies were selected by convenience sampling over the study follow-up period.

### 2.2. Ascertainment and Selection of Cases

CRC cases were selected from among Omani and non-Omani patients aged 18 years or above with histologically proven CRC (ICD-10: 18.0, 18.2 to 18.9, 19 and 20.0) who received colonoscopies at SQUH and attended daycare for colonoscopy screening during the period of the study.

Clinical information about CRC cases was extracted from the hospital information system (HIS) and medical records at the tertiary hospital. Information from medical records was compiled in abstracted evaluations. All evaluations were reviewed and scored by two clinical investigators who developed and employed a coding guide based on the International Statistical Classification of Diseases and Related Health Problems (ICD) criteria to determine if the CRC labelling was consistent with the standard international diagnostic criteria of CRC.

Interrater reliability was established among the two clinical investigators to standards of 94% agreement on the overall CRC case status. For ongoing interrater reliability checks, a random sample of records (10%) was scored independently by a reviewer experienced clinical working with CRC and did not participate in the diagnostic reviews. The percentage agreement between the raters on the final CRC case definition was found to be 98%.

### 2.3. Data Analysis

CRC prevalence estimates were calculated by dividing the number of CRC cases by a number of colonoscopies pertinent to the scope of the estimate. The prevalence estimates were reported per 100 colonoscopies. The 95% confidence intervals (95% CI) of prevalence estimates were calculated using the Poisson distribution method of binomial variables. 95% CIs were calculated using the GraphPad Prism 6.0 software. The trend in the prevalence estimates over 5 years was calculated and analyzed to identify a statistically significant increasing or decreasing trend using the Cochran–Armitage test for trend, and the cutoff for statistical significance was taken at a *P* value of 0.05.

Statistical Package for Social Sciences (SPSS) (version 24.0, IBM) was used for all statistical analyses. Ethical approval for this study was obtained from the Institutional Review Board of Sultan Qaboos University, the Medical Research Ethics Committee, at the College of Medicine and Health Sciences (MREC number 1615).

## 3. Results

Table 1 shows the distribution of the selected sociodemographic and clinical characteristics colonoscopy recipients enrolled in the study. Overall, there had been 3701 colonoscopies of which 442 (11.9%) were taken from among CRC cases. Colonoscopies obtained from males were slightly higher (1903; 51.4%). About half of the colonoscopies were conducted among patients aged below 50 years. Over the years, there has generally been a balanced distribution of total colonoscopies, with a majority being requested for by the gastroenterology clinic. The majority (36.2%) were residents at Muscat Governorate, followed by Batinah and Dakhliya (18.6% and 13.8%, respectively).

Table 1 also compares the selected characteristics among CRC cases versus “noncases.” The gender distribution was similar: around 50% among both groups. CRC cases tended to belong to the older age group compared to noncases. The distributions of each governorate of residence and year of procedure request were both comparable among CRC cases and noncases. In all comparisons, the differences were not statistically significant (*P* > 0.05).

Table 2 shows the prevalence estimates of CRC colonoscopy recipients stratified by age and gender. There were 442 CRC cases among a total of 3701 colonoscopies, yielding an overall CRC prevalence estimate of 11.9 per 100 colonoscopies (95% CI: 10.9, 13.0). The CRC prevalence per 100 colonoscopies among males (13.3; 95% CI: 11.9, 15.0) was higher than that among females (10.5; 95% CI: 9.1, 12.0). The age-specific prevalence estimates indicated that the CRC prevalence estimates increased with age, from 2.8 (95% CI: 1.9, 3.9) among participants aged below 40 years to 26.5 (95% CI 22.4, 31.2) among participants aged 70 years or more thereby yielding a 0.59% annual incremental increase ((26.5–2.8)/26.5) over the captured age range (40 years; from 38 to 78 years).

**Figure 1:**
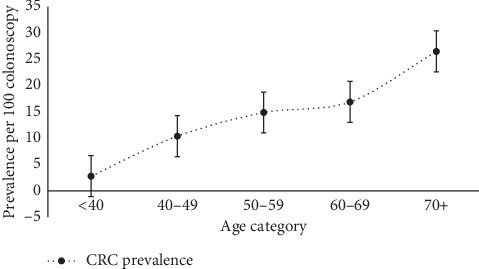
Distribution of CRC prevalence estimates over age categories, Oman, 2019.

Figure 1 depicts the distribution of CRC prevalence over increasing age categories. CRC prevalence estimates increased steadily over the increasing age categories. Overall, CRC prevalence estimates for both female and male categories increased with age.

**Figure 2:**
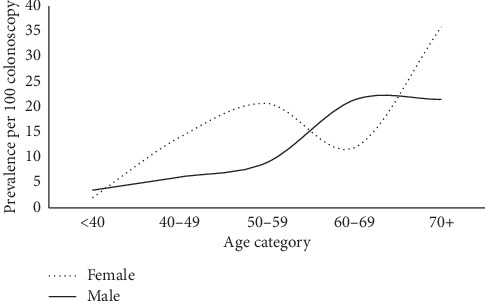
Distribution of gender-specific CRC prevalence estimates over age categories, Oman, 2019.

Figure 2 depicts the distribution of gender-specific prevalence estimates over increasing age categories. CRC prevalence among males increased steadily over age categories, reaching the highest estimate among people aged 70 years or more. CRC prevalence among females was proportionately higher than that among males overall categories except in the 60 to 69 years category.

**Figure 3:**
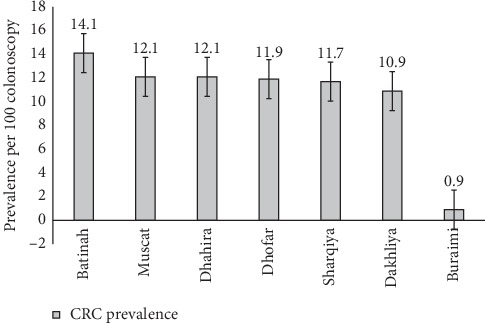
Governorate-specific estimates of the prevalence of CRC among colonoscopy recipients, Oman, 2019.

Table 3 and Figure 3 show the governorate-specific estimates of CRC prevalence among participants. The highest prevalence estimate per 100 colonoscopies was reported among participants who resided in Batinah Governorate (14.1; 95% CI 11.7, 16.9) followed by Muscat (12.1), Dhahira (12.1), Dhofar (11.9), Sharqiya (11.7), and Dakhliya (10.9). The lowest prevalence estimate was reported from Buraimi governorate (0.9; 95% CI: 0.1, 4.9).

**Figure 4:**
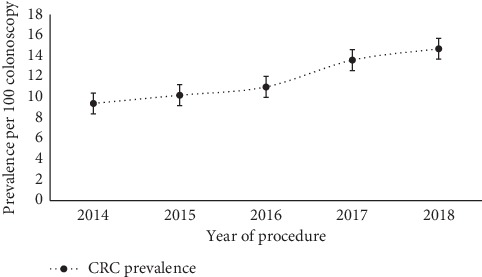
Estimates of CRC prevalence by year of diagnosis over the five-year study period, Oman, 2019.

Table 4 and Figure 4 show the distribution of CRC prevalence estimates by year of diagnosis over the five-year study period. Overall, it showed a slow-rising increase of CRC prevalence over 5 years of retrospective follow-up. The lowest CRC prevalence estimate (10.2; 95% CI: 8.0, 12.8) was reported in the year 2015, while the highest estimate (14.7; 95% CI: 12.4, 17.3) was reported in the year 2018. Therefore, the overall rise in CRC prevalence was 4.5% (1.44 folds), with an annual incremental increase of 0.9% over 5 years. Figure 4 depicts the trend of CRC prevalence estimates over a year of diagnosis.

## 4. Discussion

Contrary to trends in developed countries like Japan and Australia, where CRC incidence rates appear to be waning due to comprehensive preventive measures, evidence from economies such as those in Arab countries like Oman indicates that CRC has been on the rise over the last two decades. To promote primary prevention via colonoscopy, this cross-sectional study was conducted to estimate hospital-based CRC prevalence among colonoscopy recipients in a tertiary hospital in Oman.

The study reported an overall hospital-based CRC prevalence of 11.9 per 100 colonoscopies, considered low compared with other countries. The results of the study indicated that overall gender-specific CRC prevalence per 100 colonoscopies was higher among males compared with females by 21% (13.3 vs. 10.5). This comparative pattern between genders was similar in age-standardized incidence rates (ASRs) per 100,000 a year reported by the National Cancer Registry in Oman for the year 2015 which indicated a higher ASR among males (12.0) compared with females (9.1) by 24% [11]. The observed comparative pattern in this study was also similar to that of the US where CRC incidence was 25% higher in men than in women [19].

Over the spectrum of age categories, the study found that females were associated with a proportionately higher CRC prevalence compared with males in all age categories except among people aged 60–69 years or more where males significantly superseded them. This pattern might indicate that women in Oman are generally keener to do colonoscopies at a younger age as compared with men. It might also indicate that Omani men are more reluctant to have a colonoscopy done. The results indicated that CRC cases tended to be older over age categories compared to noncases, and age-specific CRC prevalence increased steadily by almost 10 folds with age, from 2.8 to 26.5 cases per 100 colonoscopies revealing an annual incremental increase of about 0.6% over the captured age range. The study results also indicated that the overall rise in CRC prevalence was 4.5%, with an annual incremental increase of 0.9% over 5 years. In the US, the incidence of CRC under the age of 50 has been steadily increasing at the rate of 2.1 percent per year from 1992 through 2012 [20].

The results show that the regional CRC prevalence was highest among residents in Batinah Governerate (14.1), followed by Muscat, Dhahira, and Dhofar Governorates. The lowest CRC prevalence was among the residents of the Buraimi Governorate (0.1). This pattern approximates the pattern reported by the 2015 National Cancer Registry in Oman for the ASR per 100,000 in pattern commonality as follows: Muscat (8.8), Batinah (5.8), Dakhliya (3.4), and finally Buraimi (1.9) [11]. The finding that Batinah surpasses Muscat might be affected by the catchment area and study sample size and composition. Both Batinah and Muscat are the most populated governerates in Oman. The pattern observed in this study might be explained by the fact that Muscat is the cosmopolitan capital where people have a higher tendency towards adopting a modernized, urban lifestyle, often constituting a low-fiber high-calorie diet and sedentary lifestyle compared to less urban regions. Batinah is the second-most populated governerate, and it is a growing hub for business and modern lifestyle. Mafiana et al. conducted a case-control study on 279 participants in Oman to establish baseline data for dietary and lifestyle characteristics of Omani adults diagnosed with CRC [21]. The study reported that the enrolled CRC cases consumed lower fruits and vegetables than controls and had higher overall caloric intake. The study also showed that being male, overweight, and having a family history of CRC increase the risk of CRC.

It has been reported that the regional incidence of CRC showed a 10-fold variation, and the geographic differences appear to be attributable to differences in dietary and environmental exposures that are imposed upon a background of genetically determined susceptibility [22].

Like other Arab and developing countries, CRC screening and early detection services in Oman are still considered rudimentary [23]. At present, screening is not recommended for individuals under the age of 50 unless they have inflammatory bowel disease, a history of abdominal radiation, a positive family history, or a predisposing inherited syndrome [24]. CRC is often diagnosed after the onset of symptoms, through screening colonoscopy or fecal occult blood testing in the majority of patients.

Screening of asymptomatic individuals for CRC is recommended by major societies and preventive care organizations, and it has been shown to detect asymptomatic early-stage malignancy and improve mortality [25]. Although compliance with CRC screening guidelines is steadily improving, it is still relatively low in the developing countries [23].

### 4.1. Limitations

This study is not without its limitations. First, a nonprobability sampling method (convenience sampling) was used to collect the data from one hospital and hence results cannot be generalized to the whole country. Second, the relatively small sample size may have also affected the power of the study to detect significant differences. Not all observations were statistically significant across categories in the data analysis. Finally, since the study was cross-sectional, the CRC occurrence indices were limited to prevalence only over retrospective follow-up which implied a lack of temporality and potentially reversed causality. Age-standardized incidence parameters would have been better measures of CRC occurrence with better temporality ascertainment.

## 5. Conclusion

In summary, this hospital-based cross-sectional study explored the variation in the prevalence of CRC among colonoscopy-recipients in a tertiary hospital in Oman over 5 years of retrospective follow-up. The study provides supportive evidence of a steady increase in CRC prevalence over age categories and years of follow-up and contrasted the variations of gender-specific CRC prevalence estimates over increasing age categories. The study calls for timely formulation and adoption of national CRC screening programs centered on increasing awareness of CRC and considering colonoscopy as primary prevention to respond to the steady increase in CRC prevalence in Oman and other Arab countries.

## Figures and Tables

**Table 1:** Sociodemographic and clinical characteristics of CRC cases among colonoscopy recipients, Oman, 2019.

Characteristics	Total	CRC cases	Noncases	*P* value
	(*N* = 3701)	(*N* = 442)	(*N* = 3259)	
	N (%)	N (%)	N (%)	
Gender				0.75
Female	1798 (48.6)	188 (42.5)	1610 (49.4)	
Male	1903 (51.4)	254 (57.5)	1649 (50.6)	

Age				0.91
Below 40	1123 (30.3)	31 (7.0)	1092 (33.5)	
40 to 49	768 (20.8)	80 (18.1)	688 (21.1)	
50 to 59	706 (19.1)	105 (23.8)	601 (18.4)	
60 to 69	697 (18.8)	118 (26.7)	579 (17.8)	
70 or more	407 (11.0)	108 (24.4)	299 (9.2)	

Region				0.37
Muscat	1340 (36.2)	163 (36.9)	1177 (36.1)	
Batinah	688 (18.6)	97 (21.9)	591 (18.1)	
Dakhliya	512 (13.8)	56 (12.7)	456 (14.0)	
Sharqiya	461 (12.5)	54 (12.2)	407 (12.5)	
Dhofar	318 (8.6)	38 (8.6)	280 (8.6)	
Dhahira	271 (7.3)	33 (7.5)	238 (7.3)	
Buraimi	111 (3.0)	1 (0.2)	110 (3.4)	

Year of procedure				0.21
2014	696 (18.8)	66 (14.9)	630 (19.3)	
2015	659 (17.8)	67 (15.2)	592 (18.2)	
2016	743 (20.1)	82 (18.6)	661 (20.3)	
2017	757 (20.5)	103 (23.3)	654 (20.1)	
2018	846 (22.9)	124 (28.1)	722 (22.2)	

Ordering clinic				0.24
Gastroenterology	3475 (93.9)	429 (97.1)	3046 (93.5)	
Other	226 (6.1)	13 (2.9)	213 (6.5)	

**Table 2:** Age- and gender-specific prevalence estimates of CRC among colonoscopy recipients, Oman, 2019.

Age categories	Gender categories	Total (N)	CRC (N)	Prevalence^a^ (95% CI)
Overall	Total	3701	442	11.9 (10.9, 13.0)
	Female	1798	188	10.5 (9.1, 12.0)
	Male	1903	254	13.3 (11.9, 15.0)

Below 40	Total	1123	31	2.8 (1.9, 3.9)
	Female	556	11	2.0 (1.0, 3.6)
	Male	567	20	3.5 (2.2, 5.4)

40 to 49	Total	768	80	10.4 (8.4, 12.9)
	Female	421	59	14.0 (10.9, 17.8)
	Male	347	21	6.1 (3.9, 9.2)

50 to 59	Total	706	105	14.9 (12.4, 17.8)
	Female	352	73	20.7 (16.7, 25.4)
	Male	354	32	9.0 (6.4, 12.6)

60 to 69	Total	697	118	16.9 (14.3, 20.0)
	Female	327	39	11.9 (8.7, 16.1)
	Male	370	79	21.4 (17.4, 26.0)

70 or more	Total	407	108	26.5 (22.4, 31.2)
	Female	142	51	35.9 (28.2, 44.5)
	Male	265	57	21.5 (16.8, 27.1)

^a^Prevalence is per 100 colonoscopies.

**Table 3:** Governorate-specific estimates of the prevalence of CRC among colonoscopy recipients, Oman, 2019.

Governorate	Total (N)	CRC cases (N)	Prevalence^a^ (95% CI)
Batinah	688	97	14.1 (11.7, 16.9)
Muscat	1340	163	12.1 (10.5, 14.0)
Dhahira	271	33	12.1 (8.8, 16.6)
Dhofar	318	38	11.9 (8.8, 16.0)
Sharqiya	461	54	11.7 (9.1, 14.9)
Dakhliya	512	56	10.9 (8.5, 13.9)
Buraimi	111	1	0.9 (0.1, 4.9)

**Table 4:** Estimates of CRC prevalence by year of procedure over the five-year study period, Oman, 2019.

Year of diagnosis	Total (N)	CRC cases (N)	Prevalence^a^ (95% CI)
2014	696	66	9.4 (7.5, 12.0)
2015	659	67	10.2 (8.0, 12.8)
2016	743	82	11.0 (8.9, 13.6)
2017	757	103	13.6 (11.3, 16.3)
2018	846	124	14.7 (12.4, 17.3)

## Data Availability

Data used to support this study are available on request from the corresponding author (Tel: +968 2414 3430 (office); Tel: +968 9938 3220 (mobile); Fax: +968 2441 3300 (office); E-mail: ymfarsi@squ.edu.om).
